# Omega-3 Fatty Acids Prevent Early Pancreatic Carcinogenesis via Repression of the AKT Pathway

**DOI:** 10.3390/nu10091289

**Published:** 2018-09-12

**Authors:** Yongzeng Ding, Bhargava Mullapudi, Carolina Torres, Emman Mascariñas, Georgina Mancinelli, Andrew M. Diaz, Ronald McKinney, Morgan Barron, Michelle Schultz, Michael Heiferman, Mireille Wojtanek, Kevin Adrian, Brian DeCant, Sambasiva Rao, Michel Ouellette, Ming-Sound Tsao, David J. Bentrem, Paul J. Grippo

**Affiliations:** 1 Department of Surgery, Feinberg School of Medicine, Northwestern University, Chicago, IL 60611, USA; ding.zheng@northwestern.edu (Y.D.); mullapudi.bhargav@gmail.com (B.M.); wemascarinas@gmail.com (E.M.); mrbarron01@gmail.com (M.B.); buffalosoldierms@gmail.com (M.S.); mikeheif@gmail.com (M.H.); mireillewojtanek@gmail.com (M.W.); KevinAdrian@bridgewatermcg.com (K.A.); bdecant0823@gmail.com (B.D.); dbentrem@northwestern.edu (D.J.B.); 2 Division of Gastroenterology, Department of Medicine, University of Illinois at Chicago, Chicago, IL 60612, USA; gms891@gmail.com (G.M.); amdiaz@atsu.edu (A.M.D.); mckinney@uic.edu (R.M.); s-rao@northwestern.edu (S.R.); 3 Department of Pathology, Feinberg School of Medicine, Northwestern University, Chicago, IL 60611, USA; mouellet@unmc.edu; 4 Toronto General Hospital, 200 Elizabeth St., Toronto, ON M5G 2C4, Canada; ming.tsao@uhn.ca

**Keywords:** pancreatic cancer, chemoprevention, neoplastic disease, proliferation, polyunsaturated fatty acids

## Abstract

Pancreatic cancer remains a daunting foe despite a vast number of accumulating molecular analyses regarding the mutation and expression status of a variety of genes. Indeed, most pancreatic cancer cases uniformly present with a mutation in the *KRAS* allele leading to enhanced RAS activation. Yet our understanding of the many epigenetic/environmental factors contributing to disease incidence and progression is waning. Epidemiologic data suggest that diet may be a key factor in pancreatic cancer development and potentially a means of chemoprevention at earlier stages. While diets high in ω3 fatty acids are typically associated with tumor suppression, diets high in ω6 fatty acids have been linked to increased tumor development. Thus, to better understand the contribution of these polyunsaturated fatty acids to pancreatic carcinogenesis, we modeled early stage disease by targeting mutant KRAS to the exocrine pancreas and administered diets rich in these fatty acids to assess tumor formation and altered cell-signaling pathways. We discovered that, consistent with previous reports, the ω3-enriched diet led to reduced lesion penetrance via repression of proliferation associated with reduced phosphorylated AKT (pAKT), whereas the ω6-enriched diet accelerated tumor formation. These data provide a plausible mechanism underlying previously observed effects of fatty acids and suggest that administration of ω3 fatty acids can reduce the pro-survival, pro-growth functions of pAKT. Indeed, counseling subjects at risk to increase their intake of foods containing higher amounts of ω3 fatty acids could aid in the prevention of pancreatic cancer.

## 1. Introduction

Pancreatic cancer is a highly aggressive malignancy, with a less than 10% five-year survival rate [1]. This poor prognosis is due to late diagnosis, aggressive local invasion, and poor response to conventional chemotherapy and radiotherapy [2,3]. However, recent evidence suggests that a decade or more can pass between the initial onset of early pancreatic neoplasia and the development of malignant pancreatic ductal adenocarcinoma (PDAC) [4]. This fact is an opportunity for earlier detection and treatment or chemoprevention of this disease in its initial stages, where the survival rate is considerably increased [5]. Therefore, understanding the pathogenesis of the pre-invasive stage will be essential to developing effective chemoprevention strategies for pancreatic cancer. For PDAC, there are well known epidemiologic factors that increase the risk of the disease such as age, smoking, obesity, long-standing diabetes mellitus, and chronic pancreatitis [6]. It also has been described how some cancers such as PDAC are influenced by lifestyle and environmental factors [7] and that 37% of these cases may have been prevented just by modifying these risk factors. Recent evidence suggests that diet plays a significant role in cancer etiology. In fact, the typical Western diet, with the hallmark features of a high intake of red meat, sugar, and fat, has been linked to the onset of nearly one-third of human cancers [8,9].

Fatty acids (FAs) represent the main components of dietary fat in humans and consist of hydrocarbon chains of various lengths and degrees of unsaturation that terminate with carboxylic acid groups [10]. Polyunsaturated fatty acids (PUFAs), which have multiple double bonds in their hydrocarbon chains, have been traditionally considered healthy based on their effects in cardiovascular disease. However, it is becoming more clear that they affect a wider range of diseases including type II diabetes, inflammatory disorders, and cancer [11]. The two best studied families of PUFAs are the omega-3 and omega-6 family of PUFAs. The omega-3 family is derived from α-linolenic acid (ALA: 18:3, ω-3), while the omega-6 family series originates from linoleic acid (LA: 18:2, ω-6). They are considered essential PUFAS and must be part of the diet for survival in mammals [12].

Growing evidence implicates fatty acid-induced signals as contributing to pancreatic carcinogenesis. A diet high in ω6 PUFAs is associated with greater incidence of cancer and enhanced growth of pancreatic and several other cancer types [13,14,15]. By contrast, data, including from epidemiology studies, suggest that ω3 PUFAs may have a protective role and ameliorate some advanced cancers [16]. Several in vitro studies suggest that ω3 PUFAs induce apoptosis [17,18,19], promote cell cycle arrest [17,20,21], and reduce inflammation [22,23] in pancreatic cancer, though the majority of this work was performed in cultured human cells. The mechanism by which ω3 PUFAs induce suppression of pancreatic tumorigenesis in vivo has not been clearly delineated, as there are only a few detailed reports that expand on the underlying mechanism(s) of action for how ω3 PUFAs oppose tumor development [24,25,26].

In this work we aim to unravel the mechanism by which PUFAs contribute to the development and progression of pancreatic neoplastic lesions. We have previously demonstrated that the frequency and size of pancreatic cystic papillary neoplasia (CPN) is modulated by a long-term high fatty acid diet targeted to EL-*KRAS*^G12D^ (KRAS) mice [27]. In these reports, we employed diets with 23% fat by weight with sources of ω3 (menhaden oil [27]) or ω6 (corn oil [28]) fatty acids, which is nearly equivalent to the 45% kcal diets used in this study. However, chimeric (FVB6 F1 not B6) KRAS mice were administered these two diets (which were not isocaloric) and compared to KRAS mice fed a lower fat (~10%) standard chow. Thus, caloric intake and/or chimeric background could have contributed to the effects observed in these published findings. Indeed, whether these effects on tumor development were consistent or altered following multiple durations of diet administration and the mechanism by which ω3 and ω6 PUFAs contribute to neoplastic formation/expansion were not determined. In this study, we utilized ω3- and ω6-enriched isocaloric diets to assess changes in cancer-associated mitogenic signaling. We employed more normal human pancreas cells in vitro (human pancreatic ductal epithelial (HPDE) and human pancreatic nestin-expressing (HPNE) cells) to further investigate the effects of fatty acids on these signaling pathways. These experiments affirmed the chemoprotective effect of ω3 PUFAs in pancreatic neoplasia by demonstrating that ω3 PUFAs suppress proliferation and increase apoptosis via reduced AKT phosphorylation. Moreover, we found that ω6 PUFAs accelerate lesion development, enhance proliferation, and increase levels of phosphorylated AKT (pAKT). These data demonstrate that ω3 PUFAs suppress pancreatic neoplasia by reducing AKT phosphorylation and subsequently enhancing the activity of its downstream effectors FOXO3a and BAD.

These results suggest a unique role for ω3 PUFA in the modulation of KRAS signaling through AKT. This warrants further consideration since mutant KRAS is a key initiating event of this disease, though attempts to block its activity have been elusive. Our data implicates targeting of the phosphatidylinositol-4,5-bisphosphate 3-kinase (PI3K)/AKT pathway as one primary means of blocking downstream KRAS signals which likely contribute to advanced disease.

## 2. Materials and Methods

### 2.1. Cell Culture

Human pancreatic ductal epithelial (HPDE) cells, human pancreatic ductal epithelial cells with stable expression of mutant KRAS^G12V^ (HPDE-KRAS), human pancreatic nestin-expressing (HPNE) cells, and HPNE cells with stable expression of mutant KRAS^G12V^ (HPNE-KRAS) were seeded into 6-well plates and grown to 50% confluence in keratinocyte-serum free media (SFM) with Epidermal Growth Factor (EGF) + Bovine Pituitary Extract (BPE) supplements (HPDE cells) and 10% Fetal bovine serum (FBS) in DMEM (HPNE cells). Cells were treated with bovine serum albumin (BSA), to mimic the physiologic delivery system of fats in the blood), or BSA plus either Docosahexaenoic Acid (DHA), Eicosapentaenoic acid (EPA), or Linoleic acid (LA) 20–80 µM for 24–48 h.

### 2.2. MTT Assay

Cell growth was measured using the 3-(4,5 dimethylthiazol-2yl)-2,5-diphenyl-tetrazolium bromide (MTT; Sigma-Aldrich, Co., St. Louis, MO, USA) colorimetric dye reduction method. Pancreatic cancer cells were seeded at a density of 1 × 10^4^ cells for both HPDE and HPNE cells per well in 96-well plates in complete cell culture media. After 24 h, media was replaced with serum-free medium together with indicated reagents. After 48 h, 10 μL of a 5 mg/mL MTT stock concentration was added to the medium and cultured for another 2 h. The media was then discarded and 200 μL Dimethyl sulfoxide (DMSO) was added into each well. The plate was then put on a shaker for 10 min at room temperature. The absorbances of each well were read at a wavelength of 570 nm using a spectrophotometric plate reader (Bio-TKRAS Instruments, Winooski, VT, USA).

### 2.3. Western Blotting

Cell or tissue lysates were lysed in Radioimmunoprecipitation assay (RIPA) buffer followed by needle homogenization. Equal amounts of protein (15–40 μg) were mixed with loading dye, boiled for 8 min, separated on a denaturing SDS-PAGE gel, and transferred to a Polyvinylidene difluoride (PVDF) membrane. Membranes were then blocked with 5% milk in Tris-buffered saline, 0.1% Tween 20 (TBST) for one hour before being incubated with antibodies overnight at 4 °C (pAKT, AKT, GAPDH, pBAD, pFOXO3a; 1:1000 in 5% BSA-TBST, Cell Signaling, Danvers, MA, USA). The next day membranes were washed with TBST and incubated with secondary antibody in milk at room temperature for 1 h before washing and development with Supersignal West Femto (Thermo Fisher, Waltham, MA, USA). Signals were quantified via Image J.

### 2.4. Diet Studies

KRAS mice were generated and maintained as described previously [28]. Groups of mice were administered one of three isocaloric diets containing 45% kcal from fat (see Table 1) with a combination of menhaden and safflower oils to generate ω3:ω6 ratios of 2.5:1, 1:15, and 1:125. All three diets were prepared by Research Diets (New Brunswick, NJ, Canada). All food was stored at 4 C in 5 g sealed pouches and used within 6 months to prevent/minimize fatty acid oxidation. One-month old mice were administered these diets for 7, 11, and 15 months at which points all mice were weighed and euthanized.

### 2.5. Histology

Following euthanasia, mouse pancreatic tissue was fixed in 10% buffered formalin overnight. After sectioning, slides were deparaffinized and subsequently rehydrated then stained by Hematoxylin and eosin stain (H&E) before being dehydrated and mounted. H&E stained tissue was scored for the presence of CPN lesions. Several parameters were evaluated, including the incidence (number of mice with phenotype/number of mice with genotype), frequency (total number of lesions/tissue surface area), and size (area) of precancerous lesions. The size of these lesions was determined by using a 10 mm × 10 mm grid reticle with in the eyepiece and counting the number of 1 mm^2^ grids that correspond to a specific lesion. The number of 1 mm^2^ grids is divided by the magnification of the objective lens to provide the actual area of the lesion.

### 2.6. Immunohistochemistry

After deparaffinization, the slides were heated in the microwave in Dako antigen retrieval solution ((S1699), Dako, Carpinteria, CA, USA) and then washed in Dako wash buffer (S3006). Sections were blocked with 0.5% BSA in PBS for 30 min at room temperature followed by Dako peroxidase block for 30 min. Primary anti-BrdU (Bromo-deoxyuridine) (Millipore, Burlington MA, USA) or anti-Cleaved Caspase 3 antibody (Cell Signaling, Danvers, MA, USA) was placed on sections at a concentration of 1:200 at room temperature for 1 h or overnight at 4 °C. Sections were then treated with Dako horseradish peroxidase (HRP)-linked secondary antibody (anti-rabbit or mouse IgG from Dako, Carpinteria, CA, USA) for 30 min at room temperature and developed with 3,3′-Diaminobenzidine (DAB) reagent. Immunohistochemistry for BrdU and caspase 3 were independently evaluated manually by two investigators (CT and GM), verified by ImageJ software (National Institute of Health, Bethesda, Rockville, MD, USA), and calculated as an average of the percentages of positive nuclei per total number of nuclei per field of view for each lesion evaluated.

### 2.7. Apoptosis Assay by Annexin V-FITC and Propidium Iodide (PI) Staining

Pancreatic cells (5 × 10^5^ cells/mL) were seeded and incubated in 6-well plates for 48 h with the different fatty acids treatments. After that period, the culture medium of each plate (containing cells detached during the cell death process) together with trypsinized cells were centrifuged at 200× *g* for 5 min after which pellets were resuspended in 100 μL of Annexin–V FLUOS labeling solution (PI and Annexin V–FITC) and incubated 30 min at room temperature in the dark. The percentage of cells undergoing early-stage apoptosis (Annexin V–FITC positive) or late-stage apoptosis/necrosis (Annexin V–FITC and PI positive) was measured with a Becton FC500 flow cytometer. Data were collected using BD FACS scan software, and 10,000 cells were analyzed per sample.

### 2.8. Statistical Analysis

Statistical assessments were performed using GraphPad Prism. The Student’s t-test was used for two-group comparison while differences among more than two groups were analyzed using one-way ANOVA and post hoc Bonferroni tests. The significances are shown as *p*-value < 0.05: ** *p*-value < 0.01; *** *p*-value < 0.001. In the case of Western blot analysis, one representative set of data is shown. In vitro results are presented as ± standard deviation (SD). and in vivo results are presented as ± standard error of the mean (SEM) unless otherwise noted. *N* ≥ 3 for all experiments.

## 3. Results

### 3.1. Omega-3 Enriched Diets Protect Against, Whereas ω6-Enriched Diets Accelerate Pancreatic Tumorigenesis In Vivo

In order to understand the influence of diet in the progression of the disease, we established three isocaloric diets with varying ω3:ω6 fatty acid ratios and fed them to cohorts of 1–1.5 month-old nongenic B6 and KRAS B6 mice for 7, 11, and 15 months. Each diet has 45% kcal from fat with the following ω3:ω6 fatty acid ratios: a control diet at 1:15 (similar to a standard chow), an ω3-enriched diet at 2.5:1, and an ω6-enriched diet at 1:125 (Table 1).

**Figure 1:**
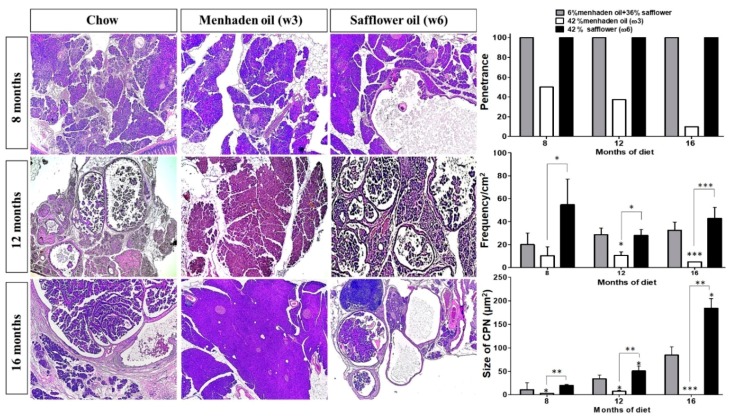
ω3-enriched diets protect against, whereas ω6-enriched diets accelerate pancreatic tumorigenesis in vivo. EL-Kras (KRAS) mice with mutant KRAS expression restricted to the pancreas acinar compartment via a rat elastase promoter were employed as a model of early pancreatic tumorigenesis. Mice were then administered either a control diet, or a diet enriched in ω3 or ω6 fatty acids, respectively, for 7, 11, or 15 months. Mice were sacrificed, tissues were sectioned and stained with H&E, and lesion frequency/penetrance and cystic papillary neoplasm (CPN) size were quantified by two blinded investigators (C.T. and G. M). * *p*-value < 0.05: ** *p*-value < 0.01; *** *p*-value < 0.001. The asterisks above each column represent the significance compared to the control diet. The significance between both diet is indicated by connecting lines.

At 8, 12, and 16 months of age, KRAS mice on standard isocaloric control or ω6-enriched diets had 100% penetrance of cystic papillary neoplasms (CPNs; resembling IPMNs or Intraductal Papillary Mucinous Neoplasms). However, mice on the ω3-enriched diet presented with less aggressive disease and a 50%, 30%, and 10% penetrance at 8, 12, and 16 months, respectively, when compared to mice on control or the ω6-enriched diet (Figure 1). KRAS mice on the ω6 diet presented with more aggressive disease than ω3 diet mice. In this sense, mice administered the ω3-enriched diet had a reduced frequency of pancreatic lesions, with a 5-, 2.5-, and 10-fold decrease at 8, 12, and 16 months respectively, compared to the other cohorts of mice on the ω6-enriched diet (Figure 1). The tumor-suppressive effect of the ω3 diet was also reflected in the overall size of neoplastic lesions, which were 10, 7, and 100 times smaller compared to the ω6-enriched diet (Figure 1) at 8, 12, and 16 months, respectively. When compared with the control diet, there were significant differences, with the lesions of the ω6-enriched diet cohorts 2, 1.5, and 2 times bigger.

**Figure 2:**
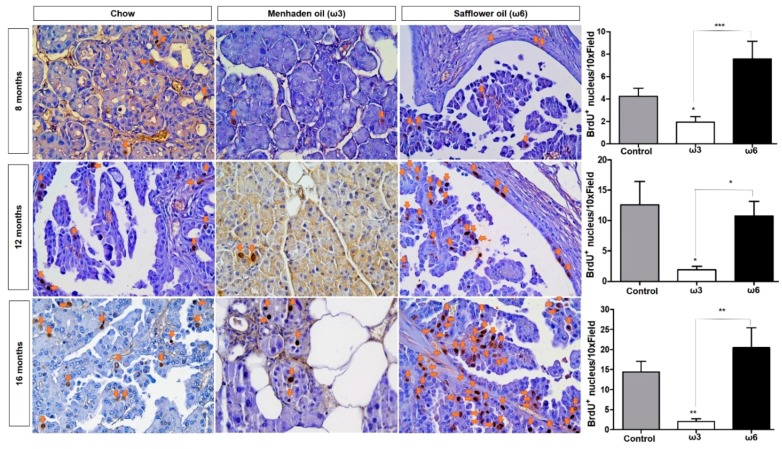
ω3-enriched diets prevent proliferation in vivo. Mice were injected with Bromo-deoxyuridine (BrdU) one hour prior to collection. Cell proliferation was assessed by immunohistochemistry to identify the incorporation of BrdU into the DNA of proliferating cells. Sections stained with BrdU antibody were scored as the number BrdU-positive nuclei per high power field by two independent researchers. * *p*-value < 0.05: ** *p*-value < 0.01; *** *p*-value < 0.001. The asterisks above each column represent the significance compared to the control diet. The significance between both diet is indicated by connecting lines.

Besides presenting with a greater number of and larger lesions, ω6 cohorts (regardless of the length of the diet) harbored increased cell proliferation measured by BrdU-positive nuclei in neoplastic tissue (Figure 2) when compared with the ω3-enriched diet. In addition, the ω3-enriched diet was able to significantly reduce proliferation in pancreas tissue of the animals compared both to control and ω6-enriched diets (Figure 2) Interestingly, the ω6-enriched diet did not show significant differences when compared to the standard chow diet.

### 3.2. Omega-3 Fatty Acids Reduce Epithelial Cell Proliferation/Viability

**Figure 3:**
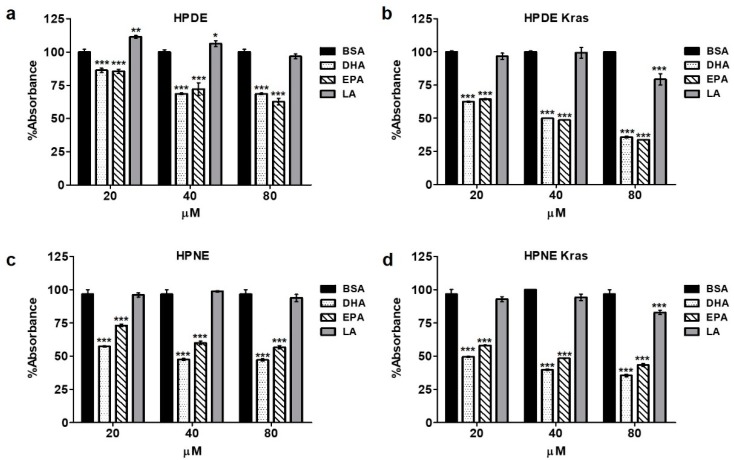
ω3 fatty acids inhibit cell viability in vitro. (**a**) Human pancreatic ductal epithelial (HPDE) cells were administered increasing concentration of either ω3 fatty acids Docosahexaenoic Acid (DHA) and Eicosapentaenoic acid (EPA), or the ω6 fatty acid Linoleic acid (LA) in vitro. Proliferation was then assessed by 3-(4,5 dimethylthiazol-2yl)-2,5-diphenyl-tetrazolium bromide (MTT) assay after 48 h, showing a similar reduction in proliferation in response to both ω3 fatty acids. (**b**–**d**) The experiment was next repeated in HPDE with stable expression of mutant KRASG12D (HPDE-KRAS), as well as human pancreatic nestin-expressing (HPNE) cells and HPNE cells with stable expression of mutant KRASG12D (HPNE-KRAS). * *p*-value < 0.05: ** *p*-value < 0.01; *** *p*-value < 0.001. The asterisk above each column represent the significance compared to the control diet. The significance between both diet is indicated by connecting lines.

Considering the opposing effects of ω3- and ω6-enriched diets on epithelial and developing neoplastic cells in mice, we explored the relationship between ω3 and ω6 fatty acids on several cellular features, including proliferation/viability, in human pancreatic cells in vitro. Consistent with our observations in KRAS mice, incubation with increasing concentrations of the ω3 fatty acids DHA or EPA reduced epithelial cell proliferation/viability in HPDE cells, generating 15, 30, and 35% reductions in the MTT colorimetric assay compared to BSA control-treated cells and 26, 30, and 31% reductions compared with the ω6 fatty acid linoleic acid (LA) at 20, 40, and 80 µM, respectively, for 48 h (Figure 3a). These observations were further accentuated in HPDE-KRAS cells, where there was significant 37, 52, and 66% reductions when compared to HPDE-KRAS cells administered BSA (Figure 3b). These effects were also observed in human pancreas nestin-expressing pancreas cells (HPNE) and HPNE-KRAS cells (Figure 3c,d), which recapitulated similar fold reduction in proliferation/viability.

### 3.3. Omega-3 Fatty Acids Inhibit AKT Phosphorylation

**Figure 4:**
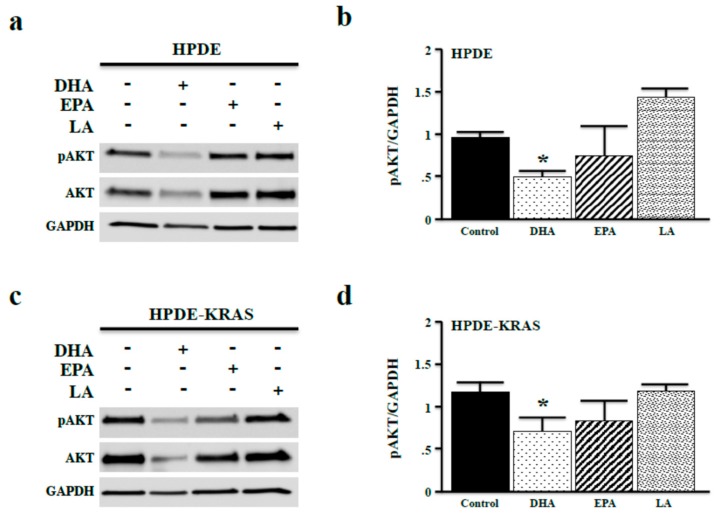
ω3 fatty acids inhibit AKT phosphorylation in vitro. (**a**,**b**) HPDE cells were again administered fixed doses of 40 µM of either the ω3 fatty acids DHA and EPA, or the ω6 fatty acid LA in vitro. After 48 h, phosphorylated AKT (pAKT) expression was measured by Western blotting and densitometry normalizing expression to GAPDH. (**c**,**d**) The experiment was repeated in HPDE-KRAS cells. (* *p* < 0.05).

One major RAS effector is PI3K, which functions to activate AKT signaling via phosphorylation (often called the PI3K/AKT pathway), leading to increased proliferation and viability. To correlate ω3-associated changes in proliferation with changes in mitogenic signaling, we investigated the effects of ω3 and ω6 fatty acids on AKT phosphorylation. After 48 h, incubation with DHA-reduced pAKT levels, consistent with reduced cell proliferation shown in Figure 3. Interestingly, only DHA reduced total levels of AKT (Figure 4a,b). In HPDE-KRAS cells, both DHA and EPA reduced pAKT expression, yet as observed in HPDE cells, only DHA reduced total levels of AKT (Figure 4c,d).

### 3.4. Omega-3 Fatty Acids Prevent FOX3a and BAD Phosphorylation In Vitro and In Vivo

**Figure 5:**
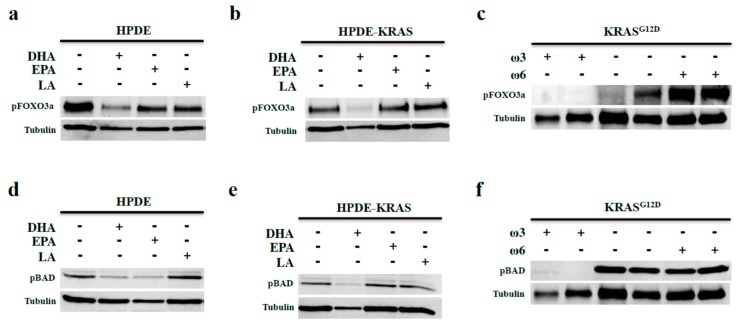
ω3 fatty acids prevent FOX3a and BAD phosphorylation in vitro and in vivo. (**a**,**b**) HPDE and HPDE-KRAS cells were incubated with fixed doses of 40 µM of the ω3 fatty acids DHA and EPA, or the ω6 fatty acid LA. Expression of the AKT-target pFOXO3a was assessed by Western blotting. (**c**) pFOXO3a expression was next evaluated in the KRAS^G12D^ mice given control, ω3-, or ω6-enriched diets for 11 months. (**d**–**f**) HPDE and HPDE-KRAS cells incubated with DHA, EPA, or LA and similarly evaluated for expression of the AKT-target pBAD. Additionally, pBAD expression was measured in KRAS mice fed control, ω3, or ω6 diets (for 11 months).

AKT-mediated phosphorylation of FOXO3a suppresses its DNA binding activity, promoting cell cycle progression [29]. As ω3 fatty acids suppress AKT phosphorylation, we assessed the effects of DHA and EPA on phosphorylation of FOXO3a. In both HPDE and HPDE-KRAS cells, incubation with DHA led to reduced FOXO3a phosphorylation (Figure 5a,b), which is consistent with our observations of increased cell cycle arrest and increased apoptosis. Moreover, mice on the ω3 diet (11 months diet) also showed reduced levels of pFOXO3a compared to that in KRAS mice fed control and ω6 diets (11-month diet). Indeed, pFOXO3a levels were elevated in ω6 diet mice (Figure 5c) compared to the control diet.

pAKT also targets the apoptotic signal BAD for phosphorylation and subsequent degradation. We hypothesized that ω3 fatty acids would decrease BAD phosphorylation similar to pFOXO3a. In HPDE cells, both DHA and EPA ω3 fatty acids led to reduced pBAD, whereas in HPDE-KRAS cells, only DHA reduced pBAD (Figure 5d,e) Similarly, pBAD was reduced in KRAS mice on the ω3 diet, yet increased in KRAS mice on the ω6 diet (11-month diet) (Figure 5f).

### 3.5. Omega-3 Fatty Acids Promote Apoptosis in Vitro and in Vivo

**Figure 6:**
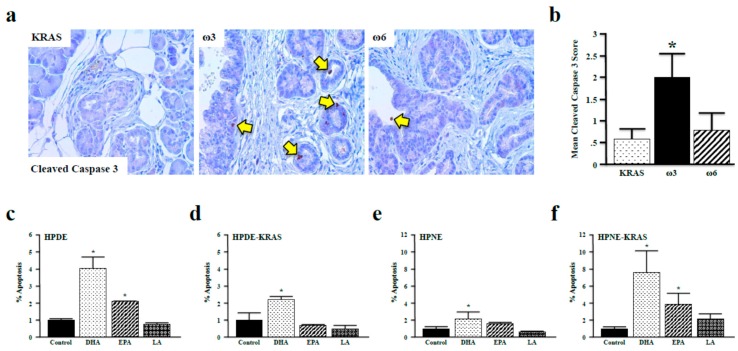
ω3 fatty acids promote apoptosis in vivo and in vitro. (**a**,**b**) Apoptosis was assessed in KRAS mice fed control, ω3, or ω6 diets (11 months) by immunohistochemistry for the surrogate marker cleaved-caspase 3. (**c**–**f**) Quantification of cell death (%) by apoptosis was performed in the HPDE, HPDE-KRAS, HPNE, and HPNE-KRAS cell lines incubated with the ω3 fatty acids DHA and EPA, or the ω6 fatty acid LA. Assays were performed in triplicate (* *p* < 0.05), and apoptosis measured via flow cytometry/Annexin–FITC assay. (* *p* < 0.05).

After determining that ω3 fatty acids suppress the phosphorylation of FOXO3a and BAD in vitro and in vivo, we investigated our hypothesis that ω3 fatty acids promote apoptosis. We first assessed cleaved-caspase 3 expression in tissues from control, ω3, and ω6 diet mice (11-month diet) because it is a surrogate marker of apoptosis in the pancreas [30]. Consistent with our previous observations, neoplastic lesions of ω3 diet mice had elevated levels cleaved-caspase 3. This was not, however, observed in ω6 diet mice (Figure 6a,b). We also incubated HPDE and HPDE-KRAS cells with the ω3 fatty acids DHA and EPA and the ω6 fatty acid LA and assessed apoptosis via flow cytometry. These results paralleled those observed in vivo, with DHA increasing apoptosis in both HPDE and HPDE-KRAS cells, and EPA increasing apoptosis solely in HPDE cells (Figure 6c,d), which was also confirmed in HPNE and HPNE-KRAS cells (Figure 6e,f).

## 4. Discussion

Pancreatic cancer remains a devastating disease, and although our understanding of the mechanisms that contribute to its etiology is improving [31], it is one of the few cancers where the incidence continues to increase. Indeed, it has been estimated that PDAC will surpass colon cancer as the second leading cause of cancer-related death in the United States in the next few years [32]. Hence, what is distinctly lacking is a better appreciation for how epigenetic events contribute to pancreatic cancer etiology. To that end, there is increasing evidence linking obesity and dietary fat to cancer progression [33]. Indeed, ω6 PUFAs are tumor-permissive in several cancers [34,35]. ω3 fatty acids, by contrast, have been shown to have a tumor suppressive effect in cancer [36,37]. However, the mechanisms by which these fatty acids impact pancreatic tumorigenesis have not been clearly delineated despite substantial epidemiological data supporting their role in the development of cancer.

Most clinical studies have correlated the intake of ω3 PUFAs with gain of weight and reduced cachexia. Wigmore et al. in 1996 performed a Phase II study where a distinct correlation between oral fish oil supplementation and reduction of cachexia in a group of pancreatic cancer patients was observed [38]. Barber et al. also suggested a role of the ω3 FA-enriched diet in preventing cachexia [39]. Many other studies have pointed to the same conclusions [40,41,42,43,44]. A few very recent prospective studies have shown a relationship between PUFA intake and digestive [45] and colon cancer risks [46,47].

Traditionally, the effects of PUFAs have been attributed to their metabolism. ω3 PUFAs can abolish the metabolism of arachidonic acid (AA) to avoid the formation of pro-inflammatory lipid mediators including a majority of prostaglandins, thromboxanes, leukotrienes, and epoxides. ω3 PUFAs, when metabolized, are the precursor of other lipid mediators with anti-inflammatory properties such as lipoxins, resolvins, protectins, and maresins [48].

In this study we have demonstrated a potential role by which ω3 FA inhibits the growth and proliferation of pancreas cells including neoplastic and cancer cells. There is a clear association between increased levels of ω3 FA and a strong decrease in the phosphorylation and activation of AKT and its downstream targets, pFOXO3a and pBAD both in vivo and in vitro, where the specific impact of EPA and DHA was determined. The implication of these findings is the potential of a non-metabolic effect of PUFAs on pancreatic cell lines and also in vivo on neoplastic lesions through a mechanism associated with changes in downstream AKT signaling. Previous studies implicated ω3 fatty acids in the negative regulation of the AKT/NF-kB cell survival pathway in breast cancer cells [49]. Also, ω3 fatty acids induced expression of the tumor suppressor PTEN [50]. There is one report that demonstrates an indirect means by which the AKT pathway is activated by the metabolism of AA via oxidation and inactivation of PTEN [51]. Previously, it has been demonstrated that ω3 FAs decreased proliferation and induced apoptosis in cultured pancreas cells (with an opposite effect through ω6 FAs) [17,20,26,27]. However, there is little information available regarding the mechanism of ω3 FA, particularly through AKT signal suppression likely independent of fatty acid metabolism, which is addressed in this study.

With regard to the AKT signaling pathway, oncogenic KRAS signals are active in 80% of PDAC patients [52] and propagate through several effector branches [53]. One of these downstream targets of KRAS is phosphatidylinositol-4,5-bisphosphate 3-kinase (PI3K) [54]. Once activated, PI3K phosphorylates phosphatidylinositol 4,5-bisphosphate (PIP2) to generate phosphatidylinositol (3,4,5)-trisphosphate (PIP3). PIP3 acts as a docking platform for other proteins to be localized in the membrane which results in their activation. One of these proteins is AKT, which promotes propagation of downstream signals related to proliferation, migration and inhibition of apoptosis [55]. Based on this information and our findings here, we have begun to investigate the contribution of PIP2 and PIP3 as mediators between PUFAs and changes in the level and activity of AKT and its downstream signals.

The contribution of these fatty acids to the AKT pathway impacting the development and progression of other cancers is becoming more clear. The ω3 fatty acids EPA and DHA have been shown to suppress the kinase activity of AKT in breast cancer cells, with DHA having a more potent inhibitory effect on AKT phosphorylation than EPA [49]. DHA and EPA also reduced the growth of LNCaP prostate cancer cells via suppression of AKT/mTOR signaling [56]. Similar findings were observed in melanoma in the Fat-1 transgenic mouse model. These mice harbor an enzyme that readily converts ω6 fatty acids to ω3 fatty acids and have upregulated PTEN expression resulting in suppressed AKT phosphorylation. Fat-1 mice were crossed to the neoplastic pancreatic tumorigenesis model p48-Cre/LSL-KRAS and fed an ω6 fatty acid-enriched diet, resulting in reduced lesion development and proliferation [57]. With regard to ω6 FAs, LA promotes pAKT-mediated proliferation in HCT116 colon cancer cells [58]. All of these findings resonate with the data we report here and confirm a distinct role of dietary fat in the formation and progression of PDAC through the AKT pathway. Indeed, this implicates targeting of the AKT pathway as a chemoprevention and/or therapy for many solid tumors including pancreatic cancer.

To this point, it has also been demonstrated that DHA supplementation increases first-line chemotherapy efficacy in patients with advanced non-small cell lung cancer [59,60]. Recently, it has been shown that nanoparticles built using bovine serum albumin (BSA)-coupled DHA and loaded with docetaxel (DTX, the standard of care for lung cancer) show increased tumor-targeting capacity, stronger anti-cancer activity in vivo, and superior efficiency for inhibiting lung cancer metastasis to bone compared with DTX treatment alone [61]. Another recent study shows the association between ω3 and ω6 FA intake and digestive cancer risks, which were modulated by other nutritional factors such as fruit and vegetables, vitamin C, and dietary fiber [45]. Hence, further investigations will be required to completely understand all the mechanisms by which fatty acids alone and in combination with other nutritional components effect cancer development. Regardless, these findings are especially encouraging in PDAC, which remains one of the most lethal cancers in dire need of effective chemoprevention and/or treatment options. It is clear that educating people and counseling patients to monitor their relative ω3:ω6 fatty acid intake may be an effective strategy in the long-term prevention of PDAC.

Combined, all these data suggest that the role of diet is critical for cancer risk and, according to our data, for suppressing early pancreatic tumorigenesis. The main implication of these results is that a diet rich in ω3 FA can abrogate the progression of pancreatic neoplasia, thus becoming a reasonable approach for PDAC chemoprevention. It also introduces an opportunity for a non-toxic approach at targeting downstream KRAS signals which may improve the efficacy of other first-line chemotherapies.

## Figures and Tables

**Table 1:** Composition of high-fat diets. Mice were fed one of three high-fat diets with the same total kcal content, 45% of which came from fat. The control diet (high-fat con) had an ω3 to ω6 ratio of 1:15, mimicking that found in a standard mouse chow. The ω3 diet had an increased ω3 to ω6 ratio (2.5:1), while the ω6 diet had a decreased ratio of 1:125.

Ingredients	ω-6	high fat con	ω-3
Menhaden Oil	-	25.5	189.5
Safflower Oil	189.5	164.0	-
Soybean Oil	13.0	13.0	13.0
Casein	200.0	200.0	200.0
Cellulose	50.0	50.0	50.0
Corn Starch	72.8	72.8	72.8
Maltodextrin	100.0	100.0	100.0
Sucrose	172.8	172.8	172.8
Mineral Mix	10.0	10.0	10.0
Dicalcium Phos	13.0	13.0	13.0
Calcium Carb	5.5	5.5	5.5
Potassium Citrate	16.5	16.5	16.5
Vitamin Mix	10.0	10.0	10.0
L-Cystine	3.0	3.0	3.0
Choline Bitartrate	2.0	2.0	2.0
**Total Amount**	858.95 g	858.84 g	858.15 g
**Caloric Intake**	4057 Kcal	4057 Kcal	4057 Kcal
Protein	23.7%	23.7%	23.7%
Fat	23.6%	23.6%	23.6%
Carbohydrates	41.4%	41.4%	41.4%
ω-3: ω-6 fa ratio	1:125	1:15	2.5:1
